# Why bother the public? A critique of Leslie Cannold’s empirical research on ectogenesis

**DOI:** 10.1007/s11017-021-09549-w

**Published:** 2021-11-30

**Authors:** Anna Smajdor

**Affiliations:** Department of Philosophy, Classics, History of Art and Ideas, University of Oslo, Oslo, Norway

**Keywords:** Ectogenesis, Abortion, Empirical research, Qualitative methods, Experimental philosophy

## Abstract

Can discussion with members of the public show philosophers where they have gone wrong? Leslie Cannold argues that it can in her 1995 paper ‘Women, Ectogenesis and Ethical Theory’, which investigates the ways in which women reason about abortion and ectogenesis (the gestation of foetuses in artificial wombs). In her study, Cannold interviewed female non-philosophers. She divided her participants into separate ‘pro-life’ and ‘pro-choice’ groups and asked them to consider whether the availability of ectogenesis would change their views about the morality of dealing with an unwanted pregnancy. The women in Cannold’s study gave responses that did not map onto the dominant tropes in the philosophical literature. Yet Cannold did not attempt to reason with her participants, and her engagement with the philosophical literature is oddly limited, focussing only on the pro-choice perspective. In this paper, I explore the question of whether Cannold is correct that philosophers’ reasoning about abortion is lacking in some way. I suggest that there are alternative conclusions to be drawn from the data she gathered and that a critical approach is necessary when attempting to undertake philosophy informed by empirical data.

## Introduction

In 1995, Leslie Cannold published a paper exploring the ways in which women respond to the idea of ectogenesis (the gestation of foetuses outside the womb), specifically as a potential alternative to abortion [1]. Ectogenesis at that time was a largely theoretical prospect. A quarter of a century later, ectogenesis is becoming increasingly scientifically plausible. In 2014, the Children’s Hospital of Philadelphia filed an international patent for ‘an extracorporeal life support system and methods of use thereof’ [2]. Subsequently, this technology has been used to sustain lamb foetuses in ‘bags’ that served as de facto artificial wombs, where they continued to develop at a comparable rate to lambs in utero [3]. In short, ectogenesis no longer looks like a merely theoretical possibility [4–7]. These new developments are happening in a context in which abortion continues to be a divisive issue (see, e.g., [8]), raising moral problems that might be resolved by the deployment of ectogenesis. Indeed, in a recent paper William Simkulet suggests that ectogenesis provides a ‘unique opportunity for moral compromise’ in the abortion debate, arguing that ‘those opposed to abortion have a *prima facie* moral obligation to pursue ectogenesis technology and provide ectogenesis for disconnected fetuses’ [9].

If ectogenesis is regarded as a potential alternative to abortion by some philosophers, this view is not shared by the medical or scientific community. Research into ectogenesis aims primarily to preserve the lives of very premature babies. Thus, even if ectogenesis becomes practicable, its status as an alternative to abortion is by no means guaranteed. Elizabeth Romanis and Claire Horn observe that treating abortion as a (moral) problem with a technological solution might serve to bolster anti-abortion positions in general, concluding that ‘the ectogenesis conversation must be regrounded in the immediate anticipated uses of the technology (neonatal intensive care) and in the immediate realities of abortion provision and reproductive inequities’ [10, p. 188]. As science continues to advance, and scholars continue to develop new and competing explanations of the relationship between ectogenesis and abortion, it is useful to look back at work such as Cannold’s and evaluate its contribution to the present debate.

A further reason for revisiting Cannold’s work is her use of empirical research. The rise of experimental philosophy has created scope for deeper understanding of the ways in which empirical methods can contribute to philosophical debate. Whether it is possible to draw normative conclusions from empirical research—as Cannold claims to do—is a contentious question.

## Overview of Cannold’s perspective

Cannold characterises the philosophical abortion debate as polarised into two camps: *severance* theorists, who believe a woman has the right to remove an unwanted foetus from her body, and *right-to-life* theorists, who believe a foetus’s right to life outweighs whatever right a woman has to bodily integrity.

Cannold suggests that ectogenesis should in theory offer a solution to both sides of the abortion debate. Severance theorists typically argue that even if the foetus has full moral status, it has no right to occupy a particular woman’s body and therefore its removal (severance) is morally permissible. Currently, the removal of a foetus invariably causes its death. But with ectogenesis, this need no longer be the case: severance could be separate from killing. Cannold holds that right-to-life theorists ought also to welcome ectogenesis, since a woman who does not wish to become a mother can have her foetus removed and transferred to an artificial womb without killing it.

Cannold’s empirical research yielded surprising results: pro-life and pro-choice women regarded ectogenesis as being an unethical alternative to abortion. On the basis of those results, Cannold criticises the ‘irrelevance of moral theory to women’s moral needs’ and suggests that philosophers do not speak ‘the same language’ as ordinary women [1, p. 63]. Philosophers, she concludes, need to change their approach.

In this paper, I critically evaluate Cannold’s conclusion by revisiting aspects of her study with an eye towards modern experimental philosophy. First, I examine problems in her treatment of the philosophical literature, including her conspicuous omission of pro-life scholarship and unsubstantiated assertion of generalised support for ectogenesis among ethicists. Second, I point to certain weaknesses in her qualitative study design, such as the failure to justify use of an all-female participant pool or take account of the methodological shortcomings of focus groups. Finally, I discuss the way in which Cannold’s empirical work functions as a kind of ‘intuition pump’, eliciting unchallenged opinions, responses, and viewpoints, which are then compared to arguments. I suggest that the lay–ethicist mismatch Cannold observes is in fact the unremarkable result of comparing unchallenged elicited intuitions with reasoned philosophical conclusions. Before turning to my critical evaluation, I start by briefly orienting Cannold’s work under the rubric of experimental philosophy.

## Is Cannold’s paper experimental philosophy?

Experimental philosophy is a fairly new name for an approach to philosophical research that has been gaining sway in recent years [11]. Cannold’s paper was published in 1995, well before the term had come into common use. For the purposes of this paper, I use the definition proposed by Stephen Stich and Kevin Tobia: ‘experimental philosophy is empirical work undertaken with the goal of contributing to a philosophical debate’ [12, p. 5]. Cannold’s research falls clearly within the parameters set by this definition. Cannold recruited participants, asked them questions, recorded their responses, evaluated the philosophical implications, drew conclusions, and published the results. To this extent, her work can be regarded as experimental philosophy.

The criticisms I level here are focussed on Cannold’s paper, rather than on experimental philosophy per se. However, they have some implications for experimental philosophers more broadly. Insofar as Cannold’s methods count as experimental philosophy, my critique could function as a cautionary note for other philosophers seeking to use the same methods. In addition to this, it could be argued that research such as Cannold’s provided some of the impetus for experimental philosophy to emerge as a specific concept in applied ethics. Cannold herself concludes from her research that philosophy needed to change. According to her, moral philosophers speak a different language from other people, their theories are irrelevant, and they fail to meet the needs of real people making moral decisions. This is a strong indictment; yet, as I will argue, the weaknesses in her methods and approach render her conclusions unconvincing. Cannold compares participants’ intuitions with philosophers’ arguments; her engagement with the philosophical literature is limited to the pro-choice severance perspective, and the mismatch that she identifies between ‘women’ and ‘philosophers’ is better characterised as a difference between some women who are not philosophers, and some women who are philosophers and also happen to be adherents of one, rather narrow, philosophical perspective on abortion.

## Cannold’s treatment of the philosophical literature

Cannold describes the philosophical landscape relating to abortion in terms of two dominant but diametrically opposed positions: ‘severance’ and ‘right to life’ (as opposed to ‘pro-choice’ and ‘pro-life’). However, problems in her characterisation of these positions—specifically, her conflation of severance with pro-life and her unsubstantiated, ad-hoc right-to-life account—significantly narrow the scope of her research and the results that she is entitled to draw from it.

Cannold gives several examples of philosophers whom she characterises as severance theorists. According to her, these philosophers hold that ‘a woman’s right “to control her body” overrides any right a fetus might have to life’, but that there is no associated right to kill any foetus that is not within a woman’s body [1, p. 56]. This view draws on the work of Judith Jarvis Thomson. Thomson’s famous violinist thought experiment describes a nonconsenting person whose body is hooked up to that of a renowned violinist with a fatal kidney ailment who will die unless he remains plugged in for the duration of his nine-month recovery period. The analogy is designed to illustrate that moral status is not the determining factor in the abortion debate [13]﻿. Rather, Thomson suggests, a woman may be justified in removing an unwanted person from her body, even if this kills him; but if removal does not kill him, she has no further right to seek his death. With respect to abortion, the implication is that the death of the foetus is justifiable only insofar as it is an unavoidable result of removal from the uterus.

However, it is important to note that not all those who support abortion rights are adherents of this so-called severance perspective. Indeed, the significance of Thomson’s argument is that it presented a new way of thinking about abortion, moving away from a focus on the moral status of the foetus towards a right to control one’s own body. Foregrounding the severance position in itself might not cause problems, provided it is made explicit that severance theorists are merely a subset of broadly pro-choice philosophers. Yet Cannold does not draw this distinction, instead allowing ‘severance theorists think *x*’ to be mistakenly extrapolated to ‘pro-choice philosophers think *x*’.

Another problem emerging from Cannold’s treatment of the literature is the absence of any detailed discussion of the right-to-life theory. In her presentation of the philosophical literature on abortion, Cannold lists several severance theorists: Mary Anne Warren, Judith Jarvis Thomson, Christine Overall, and Sissela Bok [1, fn. 9]. However, she fails to provide a single example of a philosopher who supports the right-to-life position, despite her claim that this is one of the dominant philosophical views.

This omission is puzzling in some respects, since there is a rich and diverse array of literature to explore for anyone who is interested in the arguments of those who oppose abortion. In particular, publications such as the *Linacre Quarterly*—an explicitly Catholic journal—contain a wealth of material on the subject. I mention this here because there is an interesting resonance between the ‘Catholic position on abortion’ [14] and the discussions of Cannold’s participants—both pro-life and pro-choice—as I discuss in the next section. In particular, the idea that motherhood is a special moral status that cannot be transferred to others is a key part of both. Cannold might have uncovered this affinity if she had engaged with this literature before embarking on her empirical research. Instead, she appears to have constructed her own account of the right-to-life position: Conception is when human life begins, destruction after this is murder, murder is wrong, abortion is (only) wrong because it involves the killing of the foetus, and thus if the foetus is not killed, abortion is not wrong.

One explanation for the absence from Cannold’s paper of any citation or discussion of the right-to-life position is that it is relatively common for secular philosophers to be dismissive of arguments against abortion that draw on religious perspectives, as Don Marquis has suggested [15]. My research confirms this: pro-life or religious arguments are commonly summarised and dismissed by bioethicists and moral philosophers in their own words, without engaging directly with the proponents of these views in the literature [16].

The omission of pro-life scholarship from Cannold’s discussion leads to further problems in the paper. She asserts that acceptance of ectogenesis as a solution to abortion is a ‘logically necessary commitment’ of both the severance and the right-to-life perspectives [1, p. 56]. However, because she does not engage with the right-to-life perspective, she provides no evidence that the people who adopt it are logically compelled to embrace ectogenesis. This matters for her findings because her initial claim is wrong: endorsement of ectogenesis is not entailed by the right-to-life theory.

Indeed, Cannold fails to ground either of her two initial claims in the philosophical literature, which impacts on the validity of the conclusions that she draws. To recap, her starting point before undertaking her empirical analysis is:

1. Ethicists regard ectogenesis as a solution to the problem of abortion.
2. Endorsement of ectogenesis is logically entailed by both the severance and the right-to-life theories.

Her empirical findings are:

1. Women do not regard ectogenesis as a morally acceptable alternative to abortion (regardless of their pro-life or pro-choice sympathies).
2. Women appeal to the concept of ‘being a good mother’ to explain their rejection of ectogenesis in moral terms (regardless of their pro-life or pro-choice sympathies).

And her conclusion is:

1. Ethicists have failed women by speaking in a language they do not recognise and employing concepts that are irrelevant to them.
2. This failure is indicative of a problem in moral philosophy per se.

Cannold does not cite any literature in support of either her claim that ethicists endorse ectogenesis or her claim that ectogenesis is logically entailed by the severance and right-to-life positions. With respect to the former, she may have been thinking of a paper published by Peter Singer and Deane Wells in 1984, which suggests that ectogenesis could function as a means of reconciling opposing positions in the abortion debate [17]. But aside from this paper, which she does not cite, it is not obvious that ethicists support ectogenesis as Cannold suggests.

With respect to the latter claim, it is fairly clear that right-to-life positions would not necessarily endorse ectogenesis, given the emphasis on non-transferrable maternal obligations. Neither would pro-choice positions necessarily endorse it (there is no moral imperative to save a foetus if one is convinced that it has no special moral status). I would grant, however, that the severance position seems to imply that ectogenesis would be preferable to foeticide, other things being equal.

Ultimately, Cannold’s initial claims are at best overstated and at worst simply wrong, leaving her paper with a much weaker starting point. By the time her paper was published, at least two ethicists had expressed moderately positive views on ectogenesis as a solution to the abortion debate, though Cannold does not cite them. Yet to find that some women do not endorse the views of two ethicists is in no way astonishing or groundbreaking. The significance of Cannold’s empirical findings thus appears to be far less striking than she indicates, undermining the credibility of her claim that the results of her study demonstrate the inadequacy of philosophical reasoning in this field.

## Cannold’s empirical research

I turn now to the specifics of Cannold’s qualitative empirical research and analysis. Her participants were forty-five Australian women, who were interviewed in groups of five to ten. The participants were categorised as being ‘in favour of abortion rights’ or ‘opposed to abortion rights’ (which I refer to simply as ‘pro-choice’ or ‘pro-life’ in the forthcoming discussion). The women were given vignettes to discuss, illustrating different possibilities in relation to an unwanted pregnancy. The ectogenesis vignette is given in full here:

Imagine that you are two months pregnant. You do not want to raise the child or are unable to do so and thus must decide between having an abortion or carrying the child to term and giving it up for adoption. As you are considering these options, a doctor approaches you and tells you that you have a third option. Thanks to technology, it is now possible for you to abort your fetus without killing it. Your fetus can be extracted from your body and transferred to an artificial womb where it will be grown until it is able to live outside of that artificial womb (at around nine months) then will be put up for adoption. The doctor informs you that this procedure carries no more medical risks or inconvenience to you than the traditional abortion method. Would you choose this third option? [1, p. 58]

On questioning her participants, Cannold found that both pro-life and pro-choice women rejected the prospect of ectogenesis as outlined above. The pro-life women in Cannold’s research believed that ectogenesis—the removal of the foetus from a woman’s body—is immoral, even though the foetus would not be killed. Their position is based on the idea that a morally good woman who becomes pregnant already has inalienable maternal duties towards her foetus and the child it will become [1, p. 51]. To hand the foetus over to others or a machine would represent a failure to fulfil these maternal duties.

To anyone familiar with Catholic reasoning, these ideas are not astonishing. Of course, from the Catholic point of view, killing a foetus is wrong—but it is not *all* that is wrong about abortion. Abortion represents a derogation of maternal duty, regardless of whether the foetus survives or not. The Catholic view does not sanction performing a lesser evil in order to prevent a greater one [13].^1^ So even if killing a foetus is a greater wrong than removing a foetus, it would not follow that derogating one’s maternal duty through ectogenesis is permissible as a means of avoiding the death of the foetus in abortion. Thus, like the pro-life women in Cannold’s study, the Catholic position would not endorse ectogenesis as an alternative to abortion. Cannold does not raise the subject of religion, either in her discussion of the literature or in connection with the empirical research, so it is not clear whether any of her participants were in fact Catholic or whether their reasoning simply shared these coincidental features with the Catholic position.

The pro-choice women in Cannold’s study also rejected the prospect of ectogenesis, giving very similar reasons to those given by the pro-life women. Foeticide was preferred over ectogenesis because a good mother is one who raises the child she has given birth to. For the pro-choice women, motherhood is a bond that is inalienable *except* through the death of the foetus (while for the pro-life women it is inalienable, full stop). It is precisely because ectogenesis preserves the foetus’s life that it is morally unacceptable. The choice to abort, for the pro-choice women, is construed as an exercise of maternal duty undertaken for the sake of the child*.* The point of overlap between the pro-life and pro-choice women is therefore twofold: both groups reject ectogenesis, and both groups appeal to notions of the good mother in order to explain and defend their position.

The surprising element of these results, for Cannold, is that the reasoning of these groups does not align with the dominant positions in the philosophical abortion debate. However, as I have shown, there are actually significant overlaps between the beliefs expressed by Cannold’s participants and a key strand of the pro-life philosophical literature—namely, the Catholic position. Both these women and Catholic philosophers maintain that the good mother is one who does not transfer her maternal duties to others and that these duties are inalienable (except, for the pro-choice women, through the death of the foetus). Cannold is wrong, then, when she claims that there is a ‘disjuncture’ between women and philosophers generally [1, p. 63]. Rather, in making this claim, she seems narrowly fixated on the particular observation that the pro-choice women in her study did not draw on the formal reasoning associated with severance theorists. There indeed *is* a disconnect, but I suggest this disconnect is fairly unsurprising.

The problem here is that Cannold compares the narrow *severance* position in the philosophical literature with the broad *pro-choice* position among her participants. Yet people may hold broadly pro-choice views on abortion and support them with very different kinds of argument.^2^ Pro-choice is an umbrella term that encompasses a number of possibly conflicting moral perspectives and viewpoints. Severance theory is just one of these strands of reasoning. It is not the only one, nor is it in any obvious sense the dominant strand in moral philosophy. Given this plurality, Cannold’s concern about the mismatch between pro-choice women and severance theorists seems overblown. Why should a group of pro-choice women be expected to express views matching severance theory per se, rather than any other strand of pro-choice philosophical reasoning?

A final point in this section is the question of why Cannold chose women as her participants. Of course, it is primarily women who get pregnant and have abortions. From this perspective, it makes sense to consider their opinions. However, as Cannold notes, one cannot necessarily assume that her group of forty-five Australian women offers a representative sample of all Australian women, much less all women [1, p. 58]. Indeed, in the context of experimental philosophy, it would be wrong to do so. A problem for experimental philosophers seeking to extrapolate moral positions from focus group data is the issue of dominant voices—where one or several participants dominate the discussion such that their opinion is seen or presented as the opinion of the group, at the expense of dissenting viewpoints [19]﻿. The problem is still more pronounced when morally contentious issues such as abortion are the subject of discussion. This is in part due to what is known as the social desirability bias, whereby participants will try to present themselves in the best light and provide answers that are seen to be socially desirable [20] or are otherwise influenced by wider social contexts and constructs [21]. The morally sensitive nature of abortion, in conjunction with the factors described above, means that women grouped into pro-life and pro-choice groups, if not specifically encouraged to challenge and critically evaluate their, and others’ comments, may align themselves with a dominant voice that also asserts itself as the socially desirable, or in this context, morally praiseworthy, perspective.

The challenges outlined above need not be a disaster for those want to do research of the kind that Cannold undertook. But one has to be cautious in the selection of participants, the methods of facilitation used, and the claims that can be made on the basis of the data gathered.

This caution seems to be lacking in parts of Cannold’s work. She frequently contrasts the findings from her participants qua ‘women’ with the views of ‘ethicists’. Yet Cannold neglects to observe that most of the philosophers she cites are also women (i.e., Mary Anne Warren, Judith Jarvis Thomson, Christine Overall, Sissela Bok). Given this, the dichotomy she highlights relates not to sex at all but to occupation*.* A more accurate way of expressing her concerns might be ‘female philosophers who are severance theorists use a different moral framework from women who are not philosophers, when discussing abortion’. The fact that Cannold does not discuss the reasons behind her choice either of philosophers or of participants creates uncertainty as to whether her selection of all-female philosophers is coincidental.

## Argument, intuition, and rationalisation

Cannold claims that it is ‘imperative that ethicists and the people whom they are seeking to guide—in this case women—are speaking the same language’ [1, p. 63]. She argues that the content of the women’s deliberation is ‘unfamiliar to ethicists’ and that the dominant moral theory is irrelevant ‘to women’s moral needs’ [1, p. 63]. However, there is no evidence that the women in Cannold’s research were exposed to moral theory at all. Cannold presented her participants with vignettes. Moral theories were not discussed, so it is not known how the participants would have responded to them. The fact that the views of the pro-choice women do not mirror those of the severance theorists may indicate any number of things, but to infer from this discrepancy that the philosophical perspective is inadequate seems hasty.

A key issue here is that moral philosophy is not just a static collection of facts, concepts, or theories. Rather, it is a dynamic process of reasoning. The theories that Cannold cites are the product of an iterative procedure of argument, challenge, revision, counterargument, and so forth. Of course, this kind of reasoning is not unique to philosophers; it is a part of everyday human life. Yet for this kind of reasoning to take place, one must challenge one’s thinking—or have it challenged by others.

When individuals have not considered a particular possibility before (e.g., ‘What if abortion means removal, rather than death, of the foetus’?), they may form an initial intuition only to reject it in the face of conflicting concerns as they think further, identifying inconsistencies in their reasoning or unpalatable conclusions implied. Cannold seems not to have encouraged this kind of reasoning. She grouped participants with those who held similar beliefs, minimising the opportunity for counterarguments or challenges.

It is not intuitions that form the central component of the severance or right-to-life positions, but theories and arguments.^3^ When Cannold contrasts her findings with the philosophical literature, she is not comparing like with like. Intuitions are raw in a way that arguments are not [22]. They may feed into arguments or theories, but they do not constitute these things. As far as anyone knows, the initial intuitions of philosophers could actually be exactly the same as those of Cannold’s participants—it is just that, as philosophers, they do not take their raw intuitions to be the end of the story.

In theory, the process of argument and criticism should help to avoid the risk of having people simply form a belief and then look for (moral) reasons to justify it. In practice, it is clear that both philosophers and non-philosophers have a tendency to do just this [23]. In fact, according to Jonathan Haidt, all moral argument is simply confabulation. Others have shown that philosophical training in fact strengthens people’s skills in post-hoc rationalisation [24]. In this case, perhaps there is a purity to the kind of intuitive snapshot presented in empirical research like Cannold’s.

The question of whether philosophers’ conclusions have greater value than laypeople’s intuitions is not one that I can enter into fully here. However, there are two points that are relevant whatever one’s view on rationalisation. First, if moral argument is possible at all, it should function to expose some of the elements of self-interest that are entwined with raw intuitions in moral reasoning. Second, irrespective of whether one thinks that theories and arguments present better forms of moral reasoning than intuitions, a theory or argument is simply a different phenomenon from an intuition. Thus, the problem remains that Cannold is comparing different things.

But is it really fair to characterise Cannold’s interviews as having elicited intuitions rather than arguments? Cannold emphasises in several places that the women gave moral *reasons* for their views. While Cannold does not discuss the distinction between intuition and argument explicitly, she clearly regards these moral reasons as being significant. At times, she even makes reference to a ‘moral framework’ (e.g., [1, pp. 55, 63]). Yet it is not astonishing that non-philosophers would give moral reasons for their choices. We want to feel that we are reasonably good people. We therefore couch our values and decisions in terms of moral reasons. This is part of the point I made above: self-interest pushes us towards certain conclusions and *a﻿lso* pushes us towards certain modes of justification for those conclusions. We want to make decisions that favour us and we want to feel good about them! There is nothing astonishing about this. People have found moral reasons to support their views throughout history, especially when they have an interest in the matter at stake. Slave ownership and the oppression of women are cases in point [25, 26].

Moreover, moral justifications are associated with powerful social norms. In the past, the ideal of the strong, authoritative, parental role has been invoked to justify unequal relationships between men and women and between masters and slaves. In recent times, the ideal of the good mother is so powerful that women cannot easily violate it [27]. Given this, it is less surprising that this ideal features so heavily in both the pro-choice and the pro-life positions represented in Cannold’s study. I am not suggesting here that these women’s perspectives are determined entirely by their social milieu or self-delusion, but that in the absence of critical evaluation and challenge, such views are very likely to be affected by these factors.

The import of challenge through argument is that it can make people reconsider their reasoning and identify inconsistencies. In doing so, they are able to develop their views and ultimately change their minds. It also illustrates how things that initially look simple may be far more complex. One of the severance philosophers cited by Cannold demonstrates this. Christine Overall notes that after reading and considering other viewpoints, she came to change her views on the severance position [28]. It is plausible that some of the women in Cannold’s research might have changed their views as well if they had been treated as dynamic, reasoning adults. However, Cannold does not challenge her participants. Their views, therefore, although presented as comparable to those of philosophers, may represent a different phenomenon entirely. Some critics of experimental philosophy would argue precisely this: research such as Cannold’s yields mere intuitions, whereas philosophers’ conclusions are the product of reasoning and argument [29]. If so, there is little value in eliciting the former, and to contrast them with the latter is ultimately futile.

The distinction between elicited intuitions and reasoned conclusions may explain the differences that exist between philosophers’ and laypeople’s responses to the question of whether ectogenesis presents a morally preferable alternative to abortion. In suggesting this, I do not ascribe special status to the views of philosophers over and above those of laypeople. Rather, I wish to emphasise that if intuitions are being contrasted with arguments, it is unremarkable that they do not mirror one another.

There is debate about the degree to which philosophical reflection really makes a difference to people’s conclusions. A recent study suggests this impact may be negligible. Markus Kneer et al. found that ‘people make the same judgments when they are primed to engage in careful reflection as they do in the conditions standardly used by experimental philosophers’ [30]. That is to say, people’s intuitive responses to vignettes of the sort used in Cannold’s study are expressions of deep-seated judgments. Reflection merely bolsters these responses by leading participants to produce reasons for their judgments.

Findings such as those that Cannold reports may be helpful in showing how participants react to ideas presented to them. Contrary to Kneer and colleagues’ study, though, my point in regard to Cannold is not primarily about reflection and judgment; it is about argument and conclusion. Argument necessarily involves criticism and challenge in a way that reflection does not; and while reflection gives rise to judgments, argument gives rise to conclusions. Arguments have a formal structure. Their validity depends on their logical properties. If non-philosophers present different arguments from philosophers, this difference in itself is not grounds for admonishing either group. Rather, it offers an opportunity to consider which of the two arguments is more convincing. Yet Cannold does not treat her participants’ responses as arguments. She does not evaluate them, challenge them, discuss counterarguments, or draw on existing arguments or theories. She does not do so even despite the fact that—as Cannold herself acknowledges [1, p. 61]—the pro-choice women’s perspective appears to entail that a morally good woman should kill her baby if she finds herself unable to care for it, rather than give her baby up for adoption. Perhaps this apparent entailment could be taken as a reductio of the pro-choice women’s claims regarding the relationship between good motherhood and abortion. Perhaps the women might have actually deemed foeticide preferable to adoption. At any rate, the idea that moral philosophers should change their approach in order to better accommodate these unchallenged and unrecognised possibilities seems not just misguided, but bizarre.

## Conclusion

Cannold concludes that the discrepancies between her pro-choice participants’ perspectives and those of the severance theorists demonstrate the inadequacy of philosophy in this area. As I have shown, her results do not justify such a conclusion. Cannold’s research describes some people’s responses to a specific question and indicates which moral concepts they draw on. It is not clear how, if at all, the known methodological weaknesses associated with focus groups were dealt with in the facilitation and analysis stages of her study. My critique may serve as a reminder to experimental philosophers of the challenges inherent in empirical research undertaken as a component of philosophical enquiry.

Experimental philosophers need to be sufficiently familiar with the relevant philosophical literature to be able to make meaningful claims about what is a new finding. They should be able to specify what kind of data will be elicited and what conclusions can be drawn on the basis of that data (cf. [31]). Perhaps a further challenge here for experimental philosophers, especially those working with qualitative data, would be to examine the degree to which argument, in the philosophical sense, can be accommodated in empirical research. Cannold’s failure to distinguish clearly between intuition and argument seriously limits the value of her findings.

In spite of this oversight, Cannold’s discovery of a new moral concept, ‘being a good mother’, might nevertheless have been worthwhile. However, as I have shown, this is *not* a new concept. The notion of the good mother could have been found by engaging more fully with the pro-life literature. There was no particular need to undertake empirical research to learn that such a concept exists, is used in the abortion debate, and therefore has relevance to discussions about ectogenesis. If there is no new knowledge gained from the empirical elements of a philosophical experiment, beyond that which could be established through traditional philosophical investigation, then we might ask: why bother the public at all?

## Footnotes

1 This would also be a feature of Kantian deontological reasoning, of course. Kant’s moral philosophy does not permit one to tell a lie in order to prevent a murder, for example. However, the Kantian perspective is not all that common in the abortion debate, while the Catholic one is.

2 Bonnie Steinbock gives a good overview of the philosophical variations on pro-choice abortion theory [18].

3 Intuition does, of course, play an important role in Thompson’s work, where the reader is supposed to feel intuitively that there is no obligation to allow one’s body to be ‘used’ by a renowned violinist with a fatal kidney ailment [13]. Yet Cannold does not focus on this aspect of Thompson’s work.
